# Uncertainty-aware Fourier ptychography

**DOI:** 10.1038/s41377-025-01915-w

**Published:** 2025-07-07

**Authors:** Ni Chen, Yang Wu, Chao Tan, Liangcai Cao, Jun Wang, Edmund Y. Lam

**Affiliations:** 1 Department of Electrical and Electronic Engineering, The University of Hong Kong, Hong Kong SAR, China; 2 School of Electronics and Information Engineering, Sichuan University, Chengdu, 610065 China; 3 Department of Precision Instruments, Tsinghua University, 100084 Beijing, China

**Keywords:** Imaging and sensing, Optical sensors

## Abstract

Fourier ptychography (FP) offers both wide field-of-view and high-resolution holographic imaging, making it valuable for applications ranging from microscopy and X-ray imaging to remote sensing. However, its practical implementation remains challenging due to the requirement for precise numerical forward models that accurately represent real-world imaging systems. This sensitivity to model-reality mismatches makes FP vulnerable to physical uncertainties, including misalignment, optical element aberrations, and data quality limitations. Conventional approaches address these challenges through separate methods: manual calibration or digital correction for misalignment; pupil or probe reconstruction to mitigate aberrations; or data quality enhancement through exposure adjustments or high dynamic range (HDR) techniques. Critically, these methods cannot simultaneously address the interconnected uncertainties that collectively degrade imaging performance. We introduce Uncertainty-Aware FP (UA-FP), a comprehensive framework that simultaneously addresses multiple system uncertainties without requiring complex calibration and data collection procedures. Our approach develops a fully differentiable forward imaging model that incorporates deterministic uncertainties (misalignment and optical aberrations) as optimizable parameters, while leveraging differentiable optimization with domain-specific priors to address stochastic uncertainties (noise and data quality limitations). Experimental results demonstrate that UA-FP achieves superior reconstruction quality under challenging conditions. The method maintains robust performance with reduced sub-spectrum overlap requirements and retains high-quality reconstructions even with low bit sensor data. Beyond improving image reconstruction, our approach enhances system reconfigurability and extends FP’s capabilities as a measurement tool suitable for operation in environments where precise alignment and calibration are impractical.

## Introduction

Fourier ptychographic (FP) has been implemented through various schemes including aperture-scanning^1^, camera-scanning^2–5^, mask scanning^6^, varying light source scanning^7^, and snap-shot camera array capture^8–10^. In conventional FP, each measurement captures a band-pass image of the target. These images are then computationally synthesized in the Fourier domain using phase retrieval algorithms to reconstruct the object’s complex field, while preserving the large field of view provided by the objective lens.

Despite FP’s elegant principles and versatile implementations, its practical performance is highly sensitive to the precision of the experimental setup^11–14^. Any discrepancies between the mathematical model and the physical system introduce systematic biases, leading to artifacts or distortions in the reconstructions, or even causing reconstruction failure. These issues compromise both reproducibility and confidence in the imaging results^15–17^. Addressing model-reality mismatches is challenging, often requiring sophisticated calibration procedures and deep understanding of the system’s intricacies. Precise calibration, however, is not always feasible. Low-cost FP systems, for example, may lack high-quality optical components. Furthermore, in ptychography imaging regimes beyond visible light-such as infrared, ultraviolet, X-rays, or electron imaging-additional challenges arise. These include limitations in lens quality, difficulties in generating appropriate illumination, and reduced light coherence, making accurate manual calibration extremely difficult. Such applications typically rely on advanced computational methods to tackle these challenges^18–22^. Additionally, data quality, particularly in dark-field or low-dose scenarios, depends heavily on the sensor’s dynamic range and bit depth. The multiple measurements required for FP are also susceptible to errors from light source instability, mechanical vibrations, or environmental fluctuations. These factors interact and collectively influence the overall imaging performance. Neglecting any of them can substantially degrade reconstruction quality or limit the system’s capabilities.

The research community has developed numerous digital calibration techniques for FP over the past decade, particularly for Fourier ptychographic microscopy (FPM). These include simulated annealing algorithms^23^, global positional models^24^, and frequency domain position compensation strategies^25^. To address system aberrations, pupil function recovery has been incorporated within FP to reconstruct optical aberrations introduced by the objective and tube lenses^26^. For data quality issues, advanced optimization algorithms such as global Newton’s method with well-defined cost functions^27^ have enhanced FP’s robustness against noise. Although these approaches have advanced the field significantly, they typically address challenges in isolation-digital calibration, pupil function recovery, and noise robustness are handled separately or alternately. However, these factors are often interdependent, and solving them independently leads to suboptimal outcomes. Moreover, as more uncertainties are incorporated into the forward model, analytical gradient derivation becomes increasingly complex and error-prone. Even minor model changes can render previous gradient calculations inapplicable. An alternative approach to managing FP uncertainties involves data-driven techniques using deep neural networks to learn the mapping from physical measurements to desired reconstructions, significantly improving image quality^28–30^. Despite their success in solving complex inverse problems in FP, neural network-based techniques often require specialized datasets that may not be readily accessible and may need additional training to generalize to different conditions. These approaches are also frequently criticized for their lack of interpretability. In scientific applications, where understanding underlying principles is crucial, the ability to explain how a model arrives at its decisions is essential. Traditional FP algorithms, grounded in physical principles, offer interpretability and reliability that deep learning models may lack^31^. To preserve FP’s physical significance, some approaches model the forward imaging process using feed-forward neural networks, enabling complex object information recovery during network training^32^. Additional network layers or models have been introduced to represent parameters such as pupil functions or illumination positions, which are iteratively updated during training to recover high-quality images^33,34^. However, these methods often treat system parameters independently during training, reducing their effectiveness in capturing the system’s full complexity.

It is important to recognize that while many existing methods can recover the pupil function to capture system aberrations, they often struggle to differentiate between aberrations caused by misalignment and those introduced by optical components. This limitation reduces the utility of the recovered pupil function for further analysis and calibration^35^. Accurately distinguishing between these aberration sources is crucial for precise system calibration and reconfigurability. It also enables potential co-design of the optical system and FP algorithm, facilitating new advancements in imaging performance.

In this paper, to distinguish from previous approaches, we specifically define aberrations as those arising from optical elements, separate from misalignment effects. In this work, we introduce Uncertainty-Aware Fourier Ptychography (UA-FP), a comprehensive framework that computationally addresses multiple system uncertainties including misalignment, aberrations, and data quality limitations. By modeling and jointly optimizing parameters that represent physical system uncertainties, UA-FP enhances sample reconstruction accuracy while simultaneously recovering the modeled uncertainties. This enables UA-FP to distinguish aberrations caused by misalignment from those arising from optical elements. Our proof-of-principle demonstration using FPM validates the proposed method, showing that UA-FP eliminates the need for precise alignment and calibration, enabling FP to manage complex uncertainties involving multiple interacting factors. The remainder of this paper is organized as follows: In Section 2, we present experimental results demonstrating the effectiveness of our approach. Section 3 provides a discussion and analysis of our findings. Finally, in Section 4, we detail the materials and methods used in our research, including the complete implementation of the UA-FP framework.

## Results

Our UA-FP addresses the key challenges of FP implementation by directly modeling and optimizing system uncertainties within the reconstruction process. In this section, we present experimental results that demonstrate the effectiveness of our approach compared to existing methods.

### Performance evaluation on resolution chart and biological specimens

We begin by evaluating the performance of UA-FP against several representative techniques, as summarized in Table 1. To indicate cases where aberration reconstruction is indistinguishable-i.e., where aberrations from optical elements and system misalignment cannot be accurately distinguished-we use a partially correct symbol .

**Table 1:** Imaging capabilities of the typical FP methods

	ePIE^26^	ADMM^36^	mom-FPIE^37^	mc-FPIE^38^	UA-FP (Ours)
Sample	*✓*	*✓*	*✓*	*✓*	*✓*
Aberration					*✓*
Misalignment	✗	✗	✗	*✓*	*✓*

signifies that aberrations caused by optical elements and system misalignment cannot be distinguished

Conventional methods such as embedded-PIE (ePIE)^26^, alternating direction multipliers method (ADMM)^36^, and momentum-FPIE (mom-FPIE)^37^ can reconstruct both the sample and pupil functions through alternating optimization. However, these methods are unable to correct for misalignment, leaving the recovered aberrations in the pupil plane indistinguishable. Misalignment-correction-FPIE (mc-FPIE)^38^ uses simulated annealing algorithms to address misalignment^23,25^, but due to the inaccurate modeling of the misalignment (considering only translation and rotation in the *x* − *y* plane), the recovered aberrations in the pupil plane remain indistinguishable. In our UA-FP framework, we represent system uncertainties through a comprehensive parameter set ***θ*** that includes both misalignment parameters (***θ***_illum_) and optical aberration parameters (***θ***_sys_). This integrated modeling approach allows our method to simultaneously optimize all uncertainty factors during reconstruction, enabling clear differentiation between aberrations caused by misalignment and those inherent to the optical elements themselves.

**Fig. 1:**
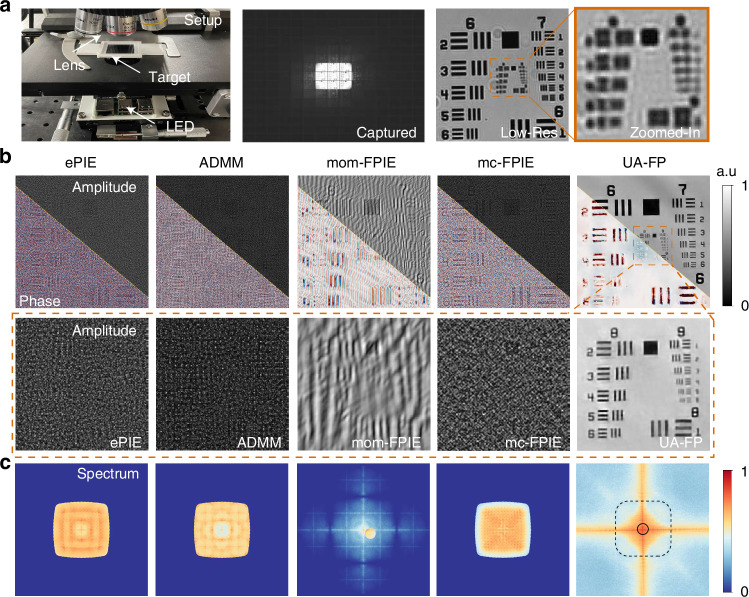
Imaging results in comparison with state-of-the-art techniques. **a** Shows our FPM setup along with the captured 15 × 15 low-resolution images. **b** Presents the reconstructed amplitude and phase, while **c** displays the reconstructed Fourier spectrum. The solid and dashed boxes represent objective lens NA and the maximum illumination NA_illum_

Our experimental setup, shown in Fig. 1a, utilizes a Raspberry Pi to control a Unicorn Hat HD 16 × 16 RGB LED array with a gap of 3.3 mm and a wavelength of 532 nm to illuminate a USAF-1951 resolution chart. The imaging system comprises an Olympus BX43 microscope equipped with a DFK 33UX273 sensor and a ×4 magnification objective lens (NA=0.1), capturing 15 × 15 low-resolution images with 8-bit Monochrome format. The LED array is located approximately 49.6 mm from the sample. To create realistic experimental conditions with inherent uncertainties, we purposely used imperfect alignment by adopting the coarse-align method^39^ without fine-align procedures. Additionally, we used identical sensor settings for both bright-field and dark-field measurements without employing high dynamic range (HDR) technology to capture the wide intensity variations. This setup results in measurements characterized by misalignment, aberrations, and low-quality data. The raw images captured, as shown in Fig. 1a, are unable to distinguish group 8 of the USAF target.

The comparative imaging results are shown in Fig. 1b, c. The amplitude and phase reconstructions in Fig. 1b demonstrate that traditional methods fail to recover the target, producing results filled with speckle noise and artifacts. In contrast, our UA-FP method successfully reconstructs the target, allowing for clear distinction of element 3 in group 9. This success stems from UA-FP’s ability to manage system imperfections simultaneously, while other methods rely on either well-calibrated systems or high-quality measurements, neither of which were available in our experimental setup. Furthermore, UA-FP demonstrates the ability to recover the Fourier spectrum beyond the synthetic spectrum, computationally inferring unrecorded information from the existing measurements. As illustrated in Fig. 1c, traditional PIE based algorithms only recover spectrum within a limited range, while UA-FP extends the Fourier spectrum to a broader scope, enabling UA-FP to achieve what we term a “computational resolution limit”.

**Fig. 2:**
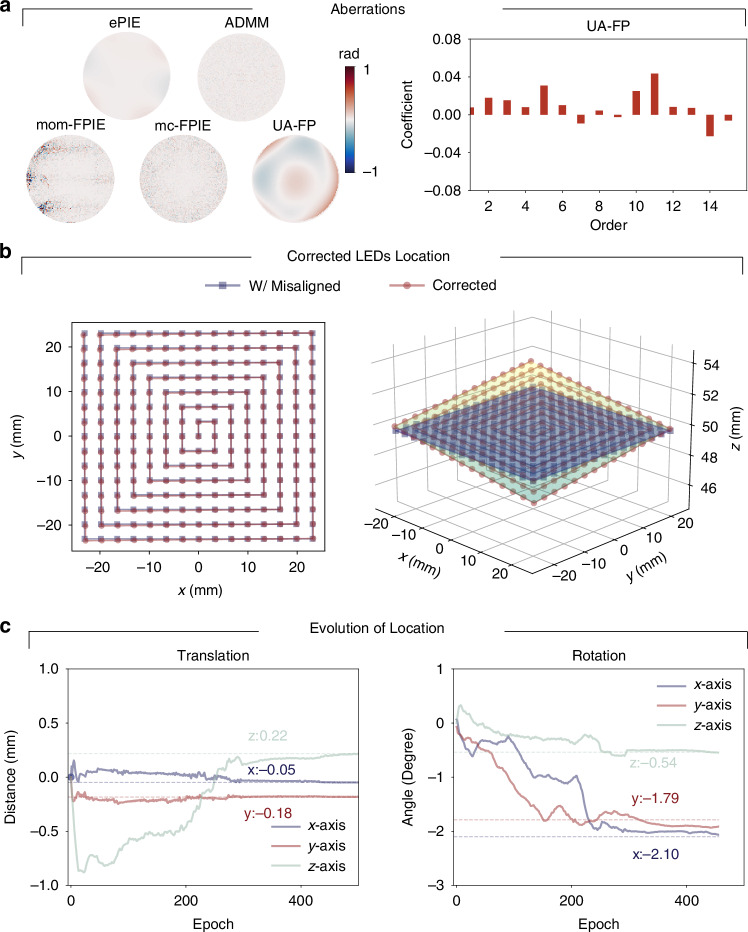
Reconstruction of system parameters. Reconstruction of system parameters including **a** aberrations and **b**, **c** misalignment. **b** Shows stereograms of the LED locations in both two dimensional (2D) and three dimensional (3D) views, before and after misalignment correction, while **c** presents the evolution of the misalignment parameter set ***θ***_illum_ during the optimization

Figure 2 demonstrates UA-FP’s capability to recover system uncertainty parameters. While all comparison methods can theoretically reconstruct pupil aberrations, they fail to achieve accurate image reconstructions with our challenging dataset. Consequently, they cannot determine the correct pupil aberration. The proposed UA-FP method successfully overcomes these limitations, as shown in Fig. 2a. The bar graph on the right side of Fig. 2a displays the coefficients of the first 15 order Zernike polynomials from the UA-FP results. This analysis reveals that the system’s aberrations are predominantly characterized by 5th-order 45^∘^ astigmatism and 11th-order spherical aberration. The correction of the misaligned LED array is illustrated in Fig. 2b, with the evolution of the ***θ***_illum_ parameters during optimization shown in Fig. 2c. During optimization, the LED array positions were initialized to their ideal locations without initial misalignment. After alignment by UA-FP, we detected translation errors of approximately 50–220 μm, which are minimal compared to the LED array pitch of 3.3 mm and thus appear negligible in the *x*-*y* view. However, the rotational analysis revealed significant deviations: approximately 2. 1^∘^ around the *x* axis, similar deviation around the *y* axis, and approximately 0.54^∘^ around the *z* axis. These results demonstrate that our UA-FP method can correct misalignment across multiple degrees of freedom without requiring additional complicated alignment techniques, highlighting its robust computational correction capabilities.

**Fig. 3:**
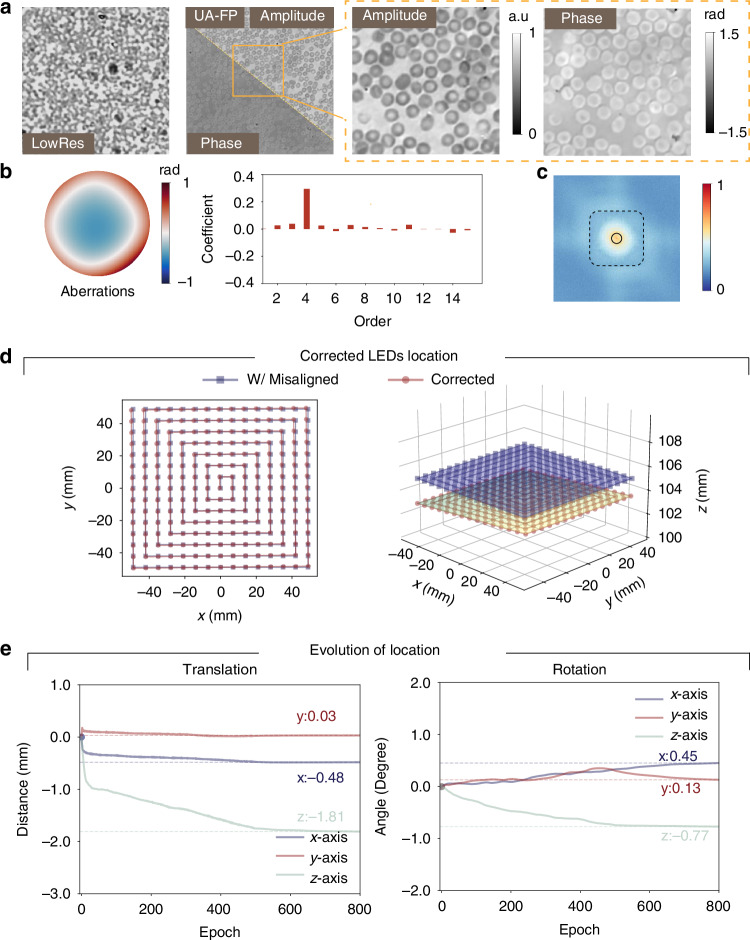
Recovery of human blood smear results. **a** Raw low-resolution image with reconstruction amplitude and phase. **b** Reconstruction aberrations and Zernike coefficients of modes. **c** Fourier spectrum, with solid and dashed boxes showing objective lens NA and maximum illumination NA_illum_. **d** 2D and 3D views of the LED array after misalignment correction. **e** Evolution of ***θ***_illum_ parameters during optimization

To evaluate the performance of UA-FP on biological samples, we modified our experimental setup to use a 15 × 15 LED array with 7 mm spacing between elements and an illumination wavelength of 520 nm. The sample was positioned approximately 107 mm from the LED array and imaged using a 4× objective lens (NA = 0.1). Using a human blood smear sample, our UA-FP method demonstrated superior reconstruction capabilities, as shown in Fig. 3. The method accurately captured the morphological features of the blood cells, particularly the characteristic central depression visible in the reconstructed phase (Fig. 3a). The reconstructed pupil function revealed significant defocusing characteristics (Fig. 3b), which is attributable to the inherent challenge of achieving precise manual focus on low-contrast biological specimens. This observation is further supported by the prominent 4th-order defocusing mode present in the Zernike coefficient analysis. Despite these challenges, the UA-FP method successfully recovered spectral information beyond the maximum illumination NA_illum_ (Fig. 3c). The LED array alignment results and the evolution of ***θ***_illum_ parameters are presented in Fig. 3d, e. The most significant misalignment in this experiment occurred in the height translation and *z*-axis rotation.

### Extended resolution capabilities beyond system limit

A particularly notable feature of UA-FP is its ability to enhance computational resolution by extending the Fourier spectrum beyond conventional limits. This capability effectively pushes the boundaries of traditional imaging, though whether mathematical approaches can truly surpass physical resolution limits remains an active area of research. Recent work has demonstrated that higher resolution can be achieved in mathematical space, particularly when incorporating well-founded prior knowledge to enhance estimation accuracy^40^. This concept of mathematical bandwidth extrapolation was first introduced by Wolter^41^ and later elaborated by Goodman^42^, who established the theoretical possibility of object reconstruction through out-of-band extrapolation. While real-world measurements invariably contain noise, our UA-FP method mitigates these adverse effects through carefully designed regularization terms and loss functions in the optimization process, enabling the recovery of information beyond conventional bandwidth limitations. To demonstrate that the information recovered beyond the synthetic spectrum is meaningful, we compared resolution changes when eliminating information outside the bandwidth.

**Fig. 4:**
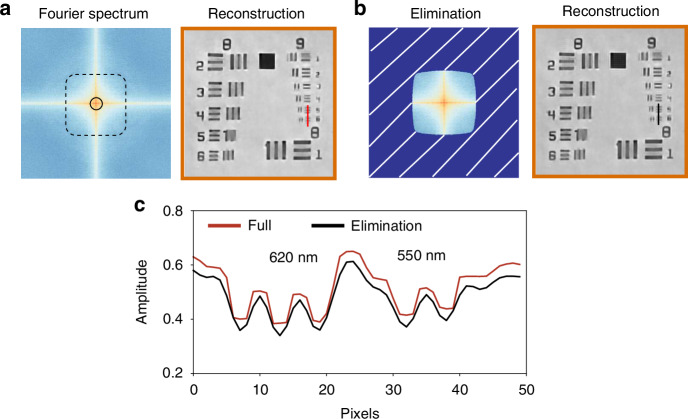
Extended imaging resolution with UA-FP. **a** Recovery full Fourier spectrum and reconstructed amplitude, and **b** Fourier spectrum by eliminating extended information and corresponding reconstructed amplitude. **c** Comparison of resolution capabilities of (**a** and **b**)

In this experiment, we used an LED array with 7 mm intervals in a 15 × 15 configuration with illumination wavelength of 520 nm. The sample-to-LED distance was 107 mm and we used a 4× objective lens (NA=0.1). Based on the theoretical resolution formula 0.61*λ*/(NA + NA_illum_), this configuration should achieve 492 nm resolution. Our results in Fig. 4c show that UA-FP achieved 550 nm resolution capability (Group 9, Element 6), which closely approaches the theoretical limit. Notably, when we eliminated information beyond the maximum illumination NA_illum_, the contrast of the target lines decreased significantly, confirming that the extended spectral information contains meaningful data. This demonstrates that UA-FP can effectively improve resolving power through mathematical extrapolation techniques.

The performance of UA-FP has been further validated using additional open-source datasets across various uncertainty levels from different imaging setups, as detailed in Supplementary S2. These results collectively demonstrate the broad applicability of our approach across different experimental conditions. To better understand the mechanisms behind this performance and provide quantitative validation with ground truth comparisons, we turn now to a detailed discussion and analysis of our findings.

## Discussion

Building on our experimental results, we now analyze the capabilities of UA-FP through controlled simulations with ground truth and perform additional evaluations to understand its robustness to various imaging challenges. These analyses provide deeper insights into the mechanisms behind UA-FP’s performance and quantify its advantages over conventional approaches.

### Quantitative validation with ground truth simulations

**Fig. 5:**
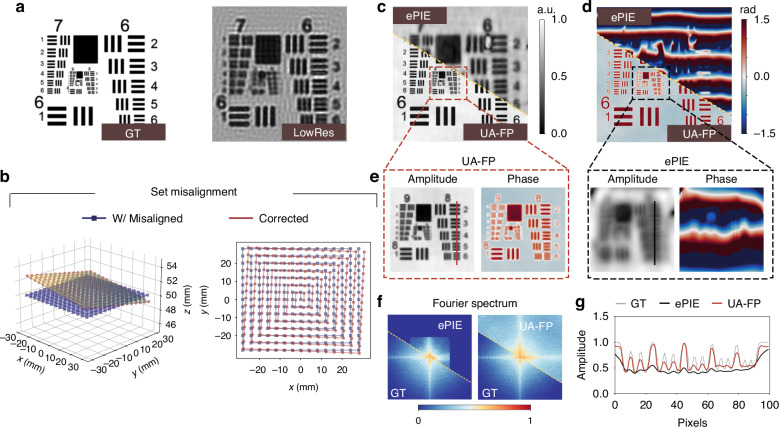
Numerical results compared with the ground truth. **a** Shows the ground truth target image alongside one of the simulated low-resolution images. **b** Depicts the introduced misalignment in the LED array. **c** and **d** Display the recovered amplitude and reconstructed phase using ePIE and UA-FP, respectively. **e** Provides a zoomed-in view of **c** and **d**, with **f** presenting a plot comparison of the lines in **e**. **g** shows the recovered Fourier spectrum of the target

While our experimental results demonstrate the practical effectiveness of UA-FP, the lack of ground truth in real-world experiments limits quantitative evaluation of reconstruction performance. To address this limitation and provide rigorous validation, we conducted numerical simulations with known system aberrations and misalignment parameters. For our simulation presented in Fig. 5, we configured a 15 × 15 square LED array with a 4 mm pitch as the light source, operating at a wavelength centered at 530 nm. The objective lens was set to 4× (NA = 0.1) magnification with a sample-to-LED distance of 50 mm and sensor pixel pitch of 3.45 μm.

The ground truth image and one of the simulated low-resolution measurements are shown in Fig. 5a. We generated controlled LED array misalignment using Eq. (3), with Fig. 5b illustrating the comparison between ideal and misaligned LED array positions. The recovered amplitude and phase using ePIE and UA-FP are displayed in Fig. 5c and d, respectively. The zoomed-in comparison in Fig. 5e clearly demonstrates that UA-FP successfully recovers both amplitude and phase with high fidelity, whereas the traditional ePIE method fails to recover the phase accurately. The line profiles in Fig. 5f further illustrate UA-FP’s superior resolving capability compared to ePIE. This performance advantage stems from UA-FP’s ability to simultaneously optimize for both aberrations and misalignment, while ePIE produces indistinguishable pupil aberration functions that conflate multiple sources of error. Notably, as shown in Fig. 5g, UA-FP recovers Fourier spectrum components beyond the synthetic spectrum-consistent with our experimental observations-achieving significantly higher reconstruction quality compared to traditional approaches.

**Fig. 6:**
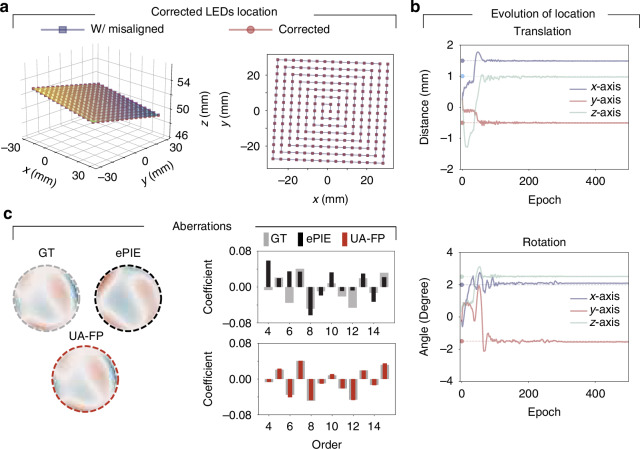
Quantitative analysis on reconstruction of the uncertainty parameters. Reconstruction of system parameters including **a**, **b** misalignment and **c** aberrations. **a** 3D and 2D views of LED array location after correction. **b** Evolution of ***θ***_illum_ parameter during the optimization. **c** Reconstructed aberrations by ePIE and UA-FP method and corresponding Zernike coefficients

Figure 6 provides detailed analysis of parameter recovery performance. The corrected LED array locations from our simulation are shown in Fig. 6a, clearly demonstrating that UA-FP accurately recovers the pre-set misaligned locations. Figure 6b illustrates the evolution of ***θ***_illum_ during the optimization process, with filled dots representing the known ground truth misalignment parameters. As optimization progresses, both translation and rotation parameters gradually converge to their true values, confirming UA-FP’s ability to correct complex misalignments with high precision. The accuracy of aberration recovery is depicted in Fig. 6c, where the Zernike coefficients for the recovered pupil function using ePIE and UA-FP are compared. The results clearly demonstrate that UA-FP accurately recovers the system aberrations with high fidelity, while ePIE’s recovered aberrations deviate significantly from the ground truth. This underscores the importance of jointly optimizing misalignment and aberration parameters to obtain physically meaningful reconstructions of both the sample and system characteristics.

To systematically evaluate UA-FP’s robustness to varying degrees of misalignment, we conducted a comprehensive analysis across different misalignment conditions. Table 2 presents numerical reconstruction results for combinations of translation (ranging from ground truth to ± 2 mm) and rotation (ranging from ground truth to ± 2.5 degrees) misalignment. The quantitative metrics reveal that as misalignment severity increases, reconstruction quality dramatically deteriorates for traditional methods such as ePIE, ADMM, and mom-FPIE, with performance drops exceeding 50% in some cases. Even mc-FPIE, which can partially address misalignment by correcting translation and rotation in the *x*-*y* plane, struggles with more complex 3D misalignments, resulting in suboptimal reconstruction quality. In stark contrast, UA-FP maintains exceptional reconstruction quality across all misalignment conditions tested. Most impressively, in cases of severe misalignment (± 2 mm translation with ± 2.5 degrees rotation), UA-FP achieves more than 135% and 78% improvement in PSNR and SSIM compared to baseline methods. This confirms UA-FP’s robust capability to handle complex misalignments in all six degrees of freedom, ensuring high-quality reconstructions even under challenging conditions.

**Table 2:** Comparison of imaging performance in the presence of both LED array misalignment and system aberrations

Translation (mm)	Rotation (degree)			Average NR (dB) / SSIM		
		ePIE^*^	ADMM	mom-FPIE	mc-FPIE	UA-FP (Ours)
Ground Truth	Ground Truth	19.42(100%) / 0.8916(100%)	**25.89(133.3%)***↑* / **0.9306(104.4%)***↑*	22.12(113.9%) / 0.9248(103.7%)	10.45(54.8%) / 0.4817(54.0%)	24.87(128.0%) / 0.9200(103.12%)
	–0.5 ~ 0.5	13.92(100%) / 0.7107(100%)	14.17(101.8%) / 0.7330(103.1%)	15.08(108.3%) / 0.7225(101.7%)	9.84(70.7%)/ 0.4557(64.1%)	**19.44(139.7%)***↑* / **0.8960(126.0%)***↑*
–0.5 ~ 0.5	–1.5 ~ 1.5	13.91(100%) / 0.7080(100%)	13.65(98.1%) / 0.7286(102.9%)	11.83(85.0%) / 0.5657(79.9%)	9.65(69.4%) / 0.4318(68.0%)	**20.18(145.1%)***↑* / **0.8990(126.9%)***↑*
	–2.5 ~ 2.5	13.88 (100%)/ 0.7023(100%)	13.73(98.9%) / 0.7239(103.0%)	9.37(67.5%) / 0.7521(107.1%)	9.51(68.5%) / 0.4505(64.1%)	**19.99(144.0%)***↑* / **0.8882(126.5%)***↑*
	–0.5 ~ 0.5	10.05(100%) / 0.5479(100%)	11.53(114.7%) / 0.6498(118.6%)	4.69(46.7%) / 0.1492(27.2%)	10.10(100.5%) / 0.4701(85.8%)	**19.60(195.0%)***↑* / **0.9020(164.6%)***↑*
–1 ~ 1	–1.5 ~ 1.5	9.94(100%) / 0.5479(100%)	11.60(116.7%) / 0.6485(118.4%)	5.67(57.0%) / 0.2164(39.5%)	10.26(103.2%) / 0.4701(85.8%)	**19.52(196.4%)***↑* / **0.9020(164.6%)***↑*
	–2.5 ~ 2.5	10.02(100%) / 0.5482(100%)	11.46(114.3%) / 0.6423(117.2%)	4.52(45.1%) / 0.1427(26.0%)	10.17(99.8%) / 0.4608(84.0%)	**19.08(190.4%)***↑* / **0.8975(163.7%)***↑*
	–0.5 ~ 0.5	9.26(100%) / 0.5072(100%)	11.07(119.6%) / 0.6213(122.5%)	3.73(23.2%) / 0.1179(100%)	9.72(104.9%) / 0.4434(87.4%)	**20.20(218.1%)***↑* / **0.8862(174.7%)***↑*
–2 ~ 2	–1.5 ~ 1.5	9.23(100%) / 0.5046(100%)	10.82(117.2%) / 0.6163(122.1%)	3.57(38.7%) / 0.1063(21.7%)	9.94(107.8%) / 0.4647(92.1%)	**21.70(235.1%)***↑* / **0.9045(179.3%)***↑*
	–2.5 ~ 2.5	9.13(100%) / 0.5019(100%)	10.44(114.4%) / 0.5917(117.8%)	3.83(41.9%) / 0.1185(23.6%)	9.69(106.1%) / 0.4639(92.3%)	**20.43(223.8%)***↑* / **0.8947(178.3%)***↑*

^*^ePIE method is chosen as the base-line method

### Robustness to noise and reduced sampling requirements

**Fig. 7:**
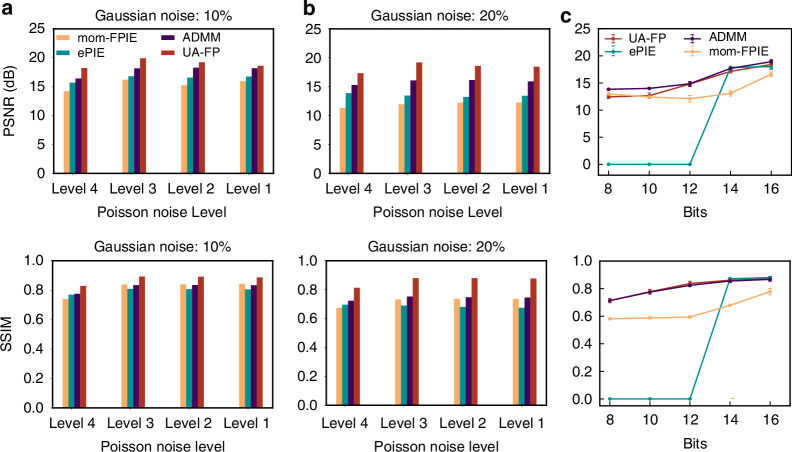
Quantitative analysis on the robustness to noise. Robustness to low-quality data was tested through anti-noise evaluations **a**, **b** and imaging quality assessments under varying sensor quantization bits **c**

Beyond misalignment correction, practical FP applications must contend with measurement noise and limited sensor bit depth. To evaluate UA-FP’s robustness to these data quality limitations, we conducted numerical simulations with varying noise levels and quantization bit depths. For our noise analysis, we considered the two predominant noise sources in FP imaging: Poisson noise for dark-field measurements (where photon counting statistics dominate) and Gaussian noise for bright-field measurements (where detector noise is more significant)^43^. Figure 7a, b shows the PSNR and SSIM of reconstructed images under various noise conditions. For dark-field images, the Poisson noise levels represent the average number of photons detected per pixel, with higher values corresponding to lower photon counts. For bright-field images, we introduced 10% and 20% Gaussian noise to simulate realistic imaging conditions. Supplementary S4 provides detailed comparison of anti-noise performance across various methods. The results consistently demonstrate UA-FP’s superior noise tolerance compared to alternative techniques. Across all noise levels tested, UA-FP maintains significantly higher reconstruction quality than competing approaches, with particularly notable advantages under severe noise conditions. Beyond noise, sensor bit depth represents another critical factor affecting data quality in practical systems. To evaluate bit depth sensitivity, we quantized the low-resolution images to 8, 10, and 12 bits, simulating sensors with different dynamic range capabilities (Fig. 7c). While traditional ePIE methods struggle to recover high-resolution images from 8-bit data, UA-FP successfully maintains high reconstruction quality even with this severe bit depth limitation. This capability is particularly valuable for low-cost FP implementations where high-bit-depth sensors may be prohibitively expensive.

**Fig. 8:**
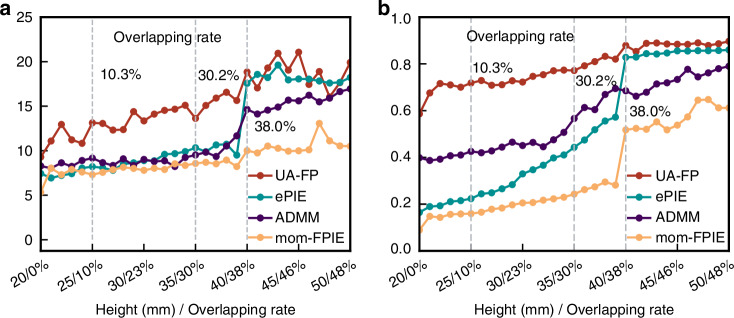
Quantitative analysis on the required measurement amount. Imaging quality assessed using the metrics PSNR **a** and SSIM **b** at different light source-to-sample distances, corresponding to varying sub-spectrum overlap ratios

An unexpected and significant finding from our analysis is that UA-FP substantially reduces the requirement for sub-spectrum overlapping-a critical parameter in traditional FP implementations. To quantify this effect, we evaluated reconstruction quality at different sample-to-LED array distances, calculating the corresponding overlapping rate based on illumination geometry (Fig. 8). The results reveal that conventional FP methods experience a dramatic performance decline when the overlapping rate falls below approximately 40%, with both PSNR and SSIM metrics showing steep drops. In contrast, UA-FP maintains stable performance at overlapping rates as low as 30%, representing a substantial relaxation of this critical system design constraint. This capability could significantly reduce the number of required measurements in practical FP implementations, accelerating acquisition speed and reducing data processing requirements.

### Broader implications and future directions

Fourier ptychography, like many computational imaging techniques, exhibits high sensitivity to various system uncertainties including misalignment, optical aberrations, and data quality limitations. The fundamental challenge in successful FP implementation lies in accurately modeling the real imaging system, which is essential for effective inverse problem solving. Our proposed UA-FP effectively addresses multiple experimental uncertainties in real-world imaging systems. By enabling simultaneous optimization of both the imaging target and system parameters, UA-FP comprehensively accounts for all factors that influence information encoding in FP implementations. Furthermore, through its incorporation of automatic differentiation, UA-FP offers enhanced flexibility in optimizing loss functions and regularization terms (details in Methods)-a significant advantage over traditional methods that are constrained to fixed loss functions.

We have demonstrated UA-FP’s effectiveness through proof-of-principle verification using FPM. Even with low-cost hardware and without fine calibration, our approach successfully performs high-quality image reconstruction by simultaneously correcting misalignment and aberrations from low-quality data. The results confirm that UA-FP achieves superior imaging performance under loose system alignments, dramatically outperforming state-of-the-art methods under challenging conditions.

Beyond improved reconstruction quality, UA-FP offers several significant practical advantages: it relaxes the sub-spectrum overlapping rate requirement-a critical constraint in traditional FP algorithms-and achieves resolution beyond the physical diffraction limit through computational spectrum extension. These capabilities substantially expand the practical utility of FP systems. The reduced requirements for system alignment and enhanced tolerance for low-quality data position UA-FP as a promising approach for expanding FP applications. This method enables FP operation beyond the visible light spectrum, where high-quality optical elements are expensive and precise alignment is challenging. Moreover, UA-FP shows significant potential for deployment in demanding environments where maintaining stable system configuration is difficult.

The ability to recover accurate system parameters provides valuable information for system analysis, calibration, and reconfiguration. These parameters also facilitate optical system and algorithm co-design, creating new possibilities for FP advancement. Furthermore, the capacity to distinguish aberrations from optical elements positions FP as a potential tool for optical component inspection and characterization^44^.

While we have demonstrated UA-FP using FPM examples, the approach extends naturally to other FP implementations such as scanning FP, addressing domain-specific challenges across various modalities. The uncertainties addressed by UA-FP-misalignment, aberrations, and data quality-are ubiquitous challenges in computational imaging systems^45–48^, making our approach broadly applicable as a general framework for uncertainty management. The underlying philosophy of UA-FP holds significant implications for computational imaging development, enabling the co-design of more robust and versatile imaging solutions across multiple domains and applications.

The quantification analysis from our simulations and experiments demonstrates that UA-FP can recover both misalignment and optical elemental aberrations while maintaining robustness to low-quality data with noise and low quantization bits. While we have presented the results and discussed their implications, the technical foundation of our approach deserves detailed examination. In the following section, we present the complete methodology behind UA-FP, explaining the principles that enable these significant improvements in FP imaging.

## Materials and methods

In this section, we present the mathematical foundation and implementation details of our UA-FP framework. After demonstrating its practical effectiveness through experimental results and numerical analysis, we now explain the technical approaches that enable these capabilities.

**Fig. 9:**
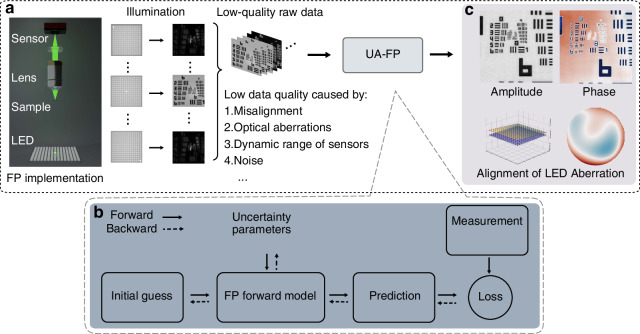
FP imaging process and the computational graph for the reconstruction. **a** FP implementation by LED array. Issues that make FP vulnerable to uncertainties include misalignment, optical element aberrations, poor quality data, and etc. **b** Proposed UA-FP, which includes differentiable FP forward model and differentiable optimization. **c** Both the imaging target and system characteristics can be recovered simultaneously

While FP implementations vary across applications, the core principles of the forward model and the sources of uncertainty remain consistent. Our methodology addresses the fundamental challenges that make FP systems vulnerable to these uncertainties. Given the significant advancements in FPM, we utilize FPM as our illustrative example, as shown in the schematic setup of Fig. 9a.

Figire 9b illustrates the schematic of the proposed UA-FP method. UA-FP integrates a forward model $f\left({{\boldsymbol{u}}}_{{\rm{obj}}},{\boldsymbol{\theta }}\right)$ that captures various uncertainties affecting the imaging system’s encoding, represented by a parameter set ***θ***, and couples it with an optimization algorithm to solve the inverse problem. In addition to reconstructing the imaging target ***u***_obj_, this approach also accounts for uncertainties that are typically unresolved by conventional methods. As a result, UA-FP reconstructions include both the imaging target and certain system characteristics in Fig. 9c. The complexity of the forward model necessitates a sophisticated computational approach, leading us to embrace the philosophy of differentiable imaging^44,49^. In UA-FP, both the forward model and the optimization process are designed to be fully differentiable, resulting in the inverse problem can be solved by automatic differentiation. The detailed implementation of UA-FP, please refer to following sections.

### Uncertainty-aware forward model of Fourier ptychography

The uncertainties in FP systems typically stem from three primary sources: misalignment, optical element aberrations, and low-quality data. While data quality is heavily influenced by factors such as sensor dynamic range, bit depth, random noise, and light source stability-making it difficult to model mathematically-we can effectively model and optimize for misalignment and optical aberrations within our framework. In FPM specifically, misalignment primarily originates from location errors in the LED array, while optical aberrations result from imperfections in the objective and tube lenses. We represent these uncertainties as a parameter set ***θ*** = {***θ***_illum_, ***θ***_sys_}, where ***θ***_illum_ corresponds to LED array location errors, and ***θ***_sys_ accounts for optical element aberrations. The *n*-th measurement ***y***_*n*_ is modeled as a function of the interaction between the imaging target ***u***_obj_ and the uncertainty parameters ***θ***, with random noise function *f*_*N*_( ⋅ ):

1 $$\begin{array}{l}{{\boldsymbol{y}}}_{n}={f}_{N}\circ f\left({{\boldsymbol{u}}}_{{\rm{obj}}},{\boldsymbol{\theta }}\right)\\ \quad\,\,={f}_{N}\circ \left\{{\left\vert {{\mathscr{F}}}^{-1}\left\{{\mathscr{F}}\left\{{{\boldsymbol{u}}}_{{\rm{obj}}}\cdot \exp \left\{j\cdot {k}_{n}\left({{\boldsymbol{\theta }}}_{{\rm{illum}}}\right)\right\}\right\}\cdot p\left({{\boldsymbol{\theta }}}_{{\rm{sys}}}\right)\right\}\right\vert }^{2}\right\}\end{array}$$

In this forward model, ***u***_obj_ represents the complex field of the imaging target. The spatial frequency in the Fourier domain corresponding to LED positions is denoted by *k*( ⋅ ), while the pupil function *p*( ⋅ ) characterizes optical aberrations present in the system. The Fourier transform operation is represented by ${\mathscr{F}}$, and the noise function *f*_*N*_( ⋅ ) encompasses both photon noise in dark-field conditions and Gaussian noise in bright-field conditions. For a detailed implementation of these components, please refer to Supplementary Section S4. This forward model structure is common across FP systems, though specific implementations of *k*( ⋅ ) and *p*( ⋅ ) may vary depending on the application context. For example, in scanning FP, *k*( ⋅ ) might correspond to scanning locations rather than LED positions, and *p*( ⋅ ) could be constant if no additional optical elements are involved. In more complex applications like remote sensing, the pupil function *p*( ⋅ ) can become highly sophisticated, particularly when addressing challenges such as atmospheric turbulence. The functions *k*( ⋅ ) and *p*( ⋅ ) represent critical components of the forward model, and their differentiability is essential for effective optimization. The following section provides a detailed explanation of both differentiable *k*( ⋅ ) and *p*( ⋅ ).

### Image reconstruction with differentiable optimization

The sophisticated forward model described above presents significant computational challenges that exceed the capabilities of traditional inverse problem solvers. These challenges arise from multiple factors: the model’s non-linear nature, the high dimensionality of unknown variables, and the inherent non-convexity of the problem. Furthermore, the system’s mixed noise profile-combining both photon and Gaussian components-adds substantial complexity to the inversion process. The interdependencies between unknown variables create additional complications, as they impede accurate gradient computation during conventional gradient descent optimization. To address these limitations, we implement a differentiable imaging framework that harnesses the power of automatic differentiation^44,49^. While automatic differentiation preserves the non-convex characteristics of the underlying problem, we effectively manage these challenges through established optimization strategies, including regularization techniques and specialized methods designed for non-convex problems. Our approach minimizes an objective function while adhering to specific physical constraints. The objective function integrates: (i) a least-squares error term to ensure data fidelity by penalizing deviations between the model and the measurement data; (ii) a regularization term acting as a soft constraint, encouraging desirable properties of both the FP system and the imaging target; and (iii) hard constraints that enforce specific physical requirements the reconstruction must satisfy. Collectively, these terms are expressed as:

2 $$\begin{array}{rc}&\mathop{{\rm{argmin}}}\limits_{{{\boldsymbol{u}}}_{{\rm{obj}}},\,{\boldsymbol{\theta }}}\quad \sum\limits_{n = 1}^{N}{\left\Vert f({{\boldsymbol{u}}}_{{\rm{obj}}},{\boldsymbol{\theta }})-{{\boldsymbol{y}}}_{n}\right\Vert }^{2}+\sum\limits_{m = 1}^{M}{\beta }_{m}{{\mathcal{R}}}_{m}({{\boldsymbol{u}}}_{{\rm{obj}}},{\boldsymbol{\theta }})\\ &\,\text{subject to}\,\quad {{\boldsymbol{u}}}_{{\rm{obj}}}\in {\Omega }_{{\boldsymbol{u}}},\quad {\boldsymbol{\theta }}\in {\Omega }_{{\boldsymbol{\theta }}}\end{array}$$

Here, ∣*f*(***u***_obj_, ***θ***) − ***y***_*n*_∣^2^ enforces data fidelity, ${{\mathcal{R}}}_{m}(\cdot )$ represents a set of soft regularization terms, with *β*_*m*_ denoting the weight assigned to each regularization term. The constraints Ω_***u***_ and Ω_***θ***_ define essential physical requirements that the system must fulfill. Ω_***u***_ specifies constraints on the objective function based on known physical properties. For instance, when working with phase distributions, these values must fall within predetermined ranges that can be adjusted to meet specific requirements. Meanwhile, Ω_***θ***_ establishes boundaries for system uncertainties, such as defining the maximum acceptable degree of misalignment in the system.

Algorithm 1 presents the pseudo-code of the UA-FP solver.

#### Algorithm 1

UA-FP Solver

**function** Reconstruct object and uncertainty parameters from ***y***_*n*_

  Initialize $\{{{\boldsymbol{u}}}_{{\rm{obj}}}^{0},\,{{\boldsymbol{\theta }}}^{0}\}$; ▹ Initialize the imaging target ***u***_obj_ and the system uncertainties ***θ***

  *f*^0^ ← *f*( ⋅ , ***θ***^0^);           ▹ Initialize the forward imaging model *f*( ⋅ )

 ** while** not converged **do**                    ▹ Iteration             

    ${\widetilde{{\boldsymbol{u}}}}^{k+1},\,{\widetilde{{\boldsymbol{\theta }}}}^{k+1}\leftarrow$ Eq. (2);            ▹ Update with gradient descent

    ${{\boldsymbol{u}}}_{{\rm{obj}}}^{k+1},\,{{\boldsymbol{\theta }}}^{k+1}\leftarrow {\Omega }_{{{\boldsymbol{u}}}_{{\rm{obj}}}}({\widetilde{{\boldsymbol{u}}}}^{k+1}),\,{\Omega }_{{\boldsymbol{\theta }}}({\widetilde{{\boldsymbol{\theta }}}}^{k+1})$;         ▹ Apply physical constraints

    *f*^*k*+1^ ← *f*( ⋅ , ***θ***^*k*+1^);                 ▹ Imaging model update

  **return** $\{{{\boldsymbol{u}}}_{{\rm{obj}}}^{K},\,{{\boldsymbol{\theta }}}^{K}\}$;                      ▹ Final iteration *K*

The objective function is optimized iteratively. During each iteration, the imaging target ***u***_obj_ and the uncertainty parameters ***θ*** are updated using gradient descent, with gradients computed through backward-mode automatic differentiation provided by PyTorch. Compared with a numerical gradient using the finite difference method, this approach avoids the problem of inaccurate gradient calculation caused by an unreasonable step size in the complex forward model. Backward-mode differentiation offers superior computational efficiency compared to forward-mode approaches^49^. This approach provides precise gradient calculations by directly interacting with the parameters of interest. Physical constraints are then applied to ensure that the updated imaging target and system parameters satisfy the required conditions. The forward model *f*( ⋅ ) is designed with inherent uncertainty parameters in its architecture. As these parameters are modified during optimization, the model must be updated accordingly to reflect these changing uncertainties. This joint optimization of the imaging target and system parameters leads to a more accurate representation of the actual FP system. For automatic differentiation to be applied effectively, the objective function itself must be differentiable, which we address in the following section.

### Differentiable forward model and loss function

We implemented the UA-FP solver using PyTorch, which allows for real-time adjustments to the forward imaging model, simultaneous optimization of multiple variables, and the integration of plug-and-play priors and physical constraints. A standard PyTorch implementation may lack differentiability across all components of the computational graph during optimization. This section identifies critical components that are non-differentiable in basic PyTorch and introduces our custom solutions to ensure end-to-end differentiability.

#### Differentiable misalignment function *k(*⋅*)*

In FP, accurately stitching the Fourier spectrum requires precise alignment of each sub-spectrum, which relates to the illumination angles of the LED array in FPM and the scanning locations in scanning FP. In FPM, the LED array can be considered as a whole, with misalignment typically caused by translations and rotations of the LED array. These misalignments are addressed through matrix operations applied to the LED coordinates. However, a naive implementation of this approach is non-differentiable due to the indexing operations involved in matrix manipulation. To address this issue, we utilize Rodrigues’ formula^50^ for a differentiable implementation. Assuming that LED location errors are represented by ***θ***_illum_ = {(*δx*, *δy*, *δz*), (*ϕ*_*x*_, *ϕ*_*y*_, *ϕ*_*z*_)}, where (*δx*, *δy*, *δz*) denotes the translation errors and (*ϕ*_*x*_, *ϕ*_*y*_, *ϕ*_*z*_) denotes the rotation errors, the misaligned LED coordinates (***x***_*mis*_, ***y***_*mis*_, ***z***_*mis*_) are expressed as

3 $$\left[\begin{array}{c}{{\boldsymbol{x}}}_{mis}\\ {{\boldsymbol{y}}}_{mis}\\ {{\boldsymbol{z}}}_{mis}\end{array}\right]=\left[\begin{array}{c}{\boldsymbol{x}}-\bar{x}\\ {\boldsymbol{y}}-\bar{y}\\ {\boldsymbol{z}}-\bar{z}\end{array}\right]\times {\left({R}_{1}\times {R}_{2}\times {R}_{3}\right)}^{T}+\left[\begin{array}{c}\bar{x}\\ \bar{y}\\ \bar{z}\end{array}\right]+\left[\begin{array}{c}\delta x\\ \delta y\\ \delta z\end{array}\right]$$

$\bar{(\cdot )}$ denotes the mean operator. The rotation matrix *R*_*n*_, where *n* = {1, 2, 3} corresponds to *x*, *y*, and *z* axes respectively, is calculated by

4 $$\begin{array}{l}{R}_{n}={\mathbb{I}}\times \left\{{\mathbb{I}}+\sin ({\phi }_{j})\cdot {M}_{CP}+\left[1-\cos ({\phi }_{n})\right]\cdot\right.\\\qquad\quad\left.({M}_{CP}\times {M}_{CP})\right\}\end{array}$$

where ${\mathbb{I}}$ is a 3 × 3 identity matrix and *M*_*CP*_ is a cross product matrix that is defined as

5 $${M}_{CP}=\left[\begin{array}{ccc}0&-{{\boldsymbol{v}}}_{n}^{3}&{{\boldsymbol{v}}}_{n}^{2}\\ {{\boldsymbol{v}}}_{n}^{3}&0&-{{\boldsymbol{v}}}_{n}^{1}\\ -{{\boldsymbol{v}}}_{n}^{2}&{{\boldsymbol{v}}}_{n}^{1}&0\end{array}\right]$$

*v*_*n*_ is a three element vector with $${{\boldsymbol{v}}}_{i}=\left\{\begin{array}{ll}1\quad \,\text{if}\,i=n\\ 0\quad \,\text{if}\,i\ne n\end{array}\right.$$. For ${{\boldsymbol{v}}}_{n}^{m}$, the sup-index *m* represents the *m*-th element of the vector ***v***_*n*_. *k*(***θ***_illum_) becomes a function of the misaligned LED coordinate as follows:

6 $$k({{\boldsymbol{\theta }}}_{{\rm{illum}}})=-\frac{2\pi }{\lambda }\frac{{{\boldsymbol{r}}}_{mis}}{\sqrt{{{\boldsymbol{r}}}_{mis}^{2}+{{\boldsymbol{z}}}_{mis}^{2}}}$$

Here, ***r***_*mis*_ represents the radial distance, calculated as $\sqrt{{x}_{mis}^{2}+{y}_{mis}^{2}}$, where *x*_*mis*_, *y*_*mis*_, and *z*_*mis*_ denote the LED element’s position in Cartesian coordinates. The variable *λ* represents the wavelength of illumination.

It is important to note that each LED location can be treated independently in scenarios where location errors occur during the manufacturing of the LED array, or in scanning FP, where each scanning step is considered independent. In these cases, *k*( ⋅ ) is a function of the misaligned LED locations or scanning locations. This increases the number of parameters that need to be optimized, making the optimization process more complex and challenging. As a result, additional priors may be required to ensure the stability and convergence of the optimization process.

#### Differentiable pupil function *p(*⋅*)*

Without considering differentiability, the pupil function in FP is typically implemented as a circular aperture with aberrations inside. The aperture size is determined by the coherent transfer function (CTF) of the imaging system, as described in Eq. (7)

7 $$\,\text{CTF}\,=\left\{\begin{array}{ll}1,\quad &{k}_{x}^{2}+{k}_{y}^{2}\le {(\text{NA}\times {k}_{0})}^{2}\\ 0,\quad &\,\text{else}\,\end{array}\right.$$

where *k*_*x*_ and *k*_*y*_ are the frequency coordinates in the *x* and *y* directions, respectively. *k*_0_ is the wave number, defined as 2*π*/*λ*. For each low-resolution image, the corresponding location of the spectrum is pre-calculated, and an identity CTF in Eq. (7) is applied to all low-resolution images. This process involves indexing operations, which lead to non-differentiable in-place operations during implementation. Furthermore, Eq. (7) represents a discontinuous function, rendering it non-differentiable both mathematically and in a programming context.

To make Eq. (7) differentiable, we address two key points: (1) Redefine the CTF as a mask matching the size of the spectrum, with the aperture shifted to the corresponding location of the low-resolution images; and (2) Use a sigmoid function to handle the discontinuity. Specifically, the sigmoid function $\frac{1}{1+\exp (-x)}$, which maps values from ( − *∞*, *∞*) to ([0, 1]), is used to approximate the traditional CTF, now shifted to the location of *k*(***θ***_illum_). This results in

8 $${\text{CTF}}_{\nabla }=\frac{1}{1+\exp \left\{\left[{({k}_{x}-{k}_{x}({{\boldsymbol{\theta }}}_{{\rm{illum}}}))}^{2}+{({k}_{y}-{k}_{y}({{\boldsymbol{\theta }}}_{{\rm{illum}}}))}^{2}\right]-{(\text{NA}\times {k}_{0})}^{2}\right\}}$$

Here, *k*_*x*_(***θ***_illum_) and *k*_*y*_(***θ***_illum_) are the wave vectors of *k*(***θ***_illum_) in the *x* and *y* axes, respectively.

The optical aberrations are modeled using Zernike polynomials within the aperture. Consequently, the phase within the aperture, *ϕ*(***θ***_sys_), is expressed in terms of Zernike coefficients ${{\boldsymbol{\theta}}}_{{\rm{sys}}}=\{{\boldsymbol{\theta}}_{0}^{0},\,\ldots ,\,{{\boldsymbol{\theta }}}_{n}^{m}\}$ and their corresponding Zernike polynomials. The phase of the aberrations, $\phi ({{\boldsymbol{\theta }}}_{n}^{m})$, is then represented as

9 $$\phi \left({{\boldsymbol{\theta }}}_{n}^{m}\right)=\exp \left\{j\times {{\boldsymbol{\theta }}}_{n}^{m}\times \sum\limits_{k = 0}^{\frac{n-m}{2}}\frac{{(-1)}^{k}\,(n-k)!}{k!\left(n+m2-k\right)!\left(n-m2-k\right)!}\ {\rho }^{n-2k}\right\}$$

where *n* and *m* are the radial order and angular meridional frequency, respectively, and *ρ* is the radius. The pupil function *p*(***θ***_sys_) is obtained by constraining the aberration phase function with the CTF_∇_:

10 $$p\left({{\boldsymbol{\theta }}}_{{\rm{sys}}}\right)={\text{CTF}}_{\nabla }\times \prod\limits_{n,\,m = 0}^{n,\,m}\phi \left({{\boldsymbol{\theta }}}_{n}^{m}\right)$$

With this approach, the output of the forward model matches the size of the input, requiring the application of a down-sampling operator *S*_down_ in the spatial domain after the inverse Fourier transform. The differentiable forward model in Eq. (2) then becomes:

11 $${{\boldsymbol{y}}}_{n}={f}_{N}\circ {S}_{{\rm{down}}}\left({\left\vert {{\mathscr{F}}}^{-1}\left({\mathscr{F}}\left({{\boldsymbol{u}}}_{{\rm{obj}}}\cdot \exp \left\{j\cdot {k}_{n}\left({{\boldsymbol{\theta }}}_{{\rm{illum}}}\right)\right\}\right)\cdot p\left({{\boldsymbol{\theta }}}_{{\rm{sys}}}\right)\right)\right\vert }^{2}\right)$$

where ${k}_{n}\left({{\boldsymbol{\theta }}}_{{\rm{illum}}}\right)$ and *p*(***θ***_sys_) are defined in Eq. (6) and Eq. (10), respectively.

From Eq. (10), it is important to note that *p*(***θ***_sys_) is also influenced by ${k}_{n}\left({{\boldsymbol{\theta }}}_{{\rm{illum}}}\right)$, reflecting the internal interactions between the parameters to be optimized. These interactions complicate the optimization process, making conventional optimization methods unsuitable for this problem.

#### Loss function

The general minimization problem in Eq. (2) can be effective even without regularization and constraints if the data quality is high, such as when using HDR imaging or varying exposure times during measurement, as is often done with conventional techniques. However, these approaches can make measurement and data processing more complex and time-consuming. Our approach aims to alleviate this burden by leveraging computational methods rather than relying on specialized data measurement techniques.

We design our objective function for scenarios where data quality is low, the system is misaligned, and the optical elements are aberrated. To address these challenges, we incorporate total variation (TV) and feature-based regularization into our objective function. Specifically, we optimize the following objective function:

12 $$\begin{array}{l}\mathop{{\rm{argmin}}}\limits_{{{\boldsymbol{u}}}_{{\rm{obj}}},\,{{\boldsymbol{\theta }}}_{{\rm{sys}}},\,{{\boldsymbol{\theta }}}_{{\rm{illum}}}}\quad \sum\limits_{n = 1}^{N}{\left\Vert f({{\boldsymbol{u}}}_{{\rm{obj}}},{{\boldsymbol{\theta }}}_{{\rm{sys}}},{{\boldsymbol{\theta }}}_{{\rm{illum}}})-{{\boldsymbol{y}}}_{n}\right\Vert }^{2}+{\beta }_{1}{{\mathcal{R}}}_{TV}({{\boldsymbol{u}}}_{{\rm{obj}}})\\ \qquad\qquad\qquad +\,{\beta }_{2}\sum\limits_{n = 1}^{N}| \Phi [f({{\boldsymbol{u}}}_{{\rm{obj}}},\,{{\boldsymbol{\theta }}}_{{\rm{sys}}},\,{{\boldsymbol{\theta }}}_{{\rm{illum}}})]-\Phi [{{\boldsymbol{y}}}_{n}]|\end{array}$$

In this formulation, ${{\mathcal{R}}}_{TV}(\cdot )$ represents TV regularization, defined as $\frac{1}{N}\sum\nolimits_{n = 1}^{N}\sqrt{| {\nabla }_{x}{{\boldsymbol{u}}}_{{\rm{obj}}}{| }^{2}+| {\nabla }_{y}{{\boldsymbol{u}}}_{{\rm{obj}}}{| }^{2}+\tau | }$, where ∇_*x*_ and ∇_*y*_ denotes the first-order gradient operator in *x* and *y* axis, respectively, and *τ* is a small constant (approximately 10^−8^) to prevent numerical instabilities. This regularization helps reduce noise and unwanted artifacts in the reconstructed image. The second regularization term focuses on capturing the key features of *f*( ⋅ ) and ***y***_*n*_. Inspired by error-laxity Fourier ptychographic iterative engine (ELFPIE)^16,51^, we apply a classical image feature extraction technique using the first-order difference operator. Specifically, we convolve with kernels [0, − 1, 1] and [0, −1, 1]^*T*^ along the *x* and *y* axes, respectively. This method enhances the reconstruction by incorporating both TV and feature regularization. The significance of the feature regularization term is discussed in detail in Supplementary S3. To ensure the differentiability of the objective function, the feature-extraction term in Eq. (12) must also be differentiable. We accomplish this by using a smooth approximation of the *ℓ*_1_ norm if there are numerous singular points.

## Supplementary information

Supplementary Information for Uncertainty-Aware Fourier Ptychography

## Data Availability

The data and code are available upon reasonable request to the corresponding author Ni Chen.
