# Supplementary material for: Uncertainty-aware Fourier ptychography
Source: Light Sci Appl. 2025 Jul 7;14:236. doi: 10.1038/s41377-025-01915-w (PMC12234902; doi:10.1038/s41377-025-01915-w)
Supplement: Supplementary file 1 — Supplementary Information for Uncertainty-Aware Fourier Ptychography [file 41377_2025_1915_MOESM1_ESM.pdf]

Supplementary Information for Uncertainty-Aware Fourier Ptychography

Ni Chen[1,†,*], Yang Wu[2,†], Chao Tan[2], Liangcai Cao[3], Jun Wang[2,*], and Edmund Y. Lam[1,*]

[1]Department of Electrical and Electronic Engineering, The University of Hong Kong, Hong Kong SAR, China
[2]School of Electronics and Information Engineering, Sichuan University, Chengdu 610065, China
[3]Department of Precision Instruments, Tsinghua University, Beijing 100084, China
[†]Equal contribution
[*]Corresponding authors: nichen@eee.hku.hk, elam@eee.hku.hk, jwang@scu.edu.cn

S1. Results with USAF target

We present additional experimental results to validate our method. In this experimental setup, the LED array consists of 15×15 elements with $7\,\mathrm{mm}$ spacing between elements and an illumination wavelength of $520\,\mathrm{nm}$. The sample is positioned approximately $107\,\mathrm{mm}$ from the LED array and imaged using a $4\times$ objective lens (NA 0.1). For NA enhancement, we provide both theoretical and practical enhancement results based on the experimental parameters. According to the theoretical resolution formula $0.61\lambda/(\mathrm{NA}+\mathrm{NA}_{\mathrm{illum}})$, the system should achieve $492\,\mathrm{nm}$ resolution theoretically, with the illumination $\mathrm{NA}_{\mathrm{illum}}$ calculated as 0.54. Figure S1 presents the reconstruction of the resolution target using this system. The experimental results demonstrate resolution capable of distinguishing element 6 of group 9 ($550\,\mathrm{nm}$), closely matching the theoretical value. This indicates that the maximum illumination $\mathrm{NA}_{\mathrm{illum}}$ achieved experimentally reaches 0.47. Thus, the improvement in NA exceeds $4\times$ the objective lens NA.

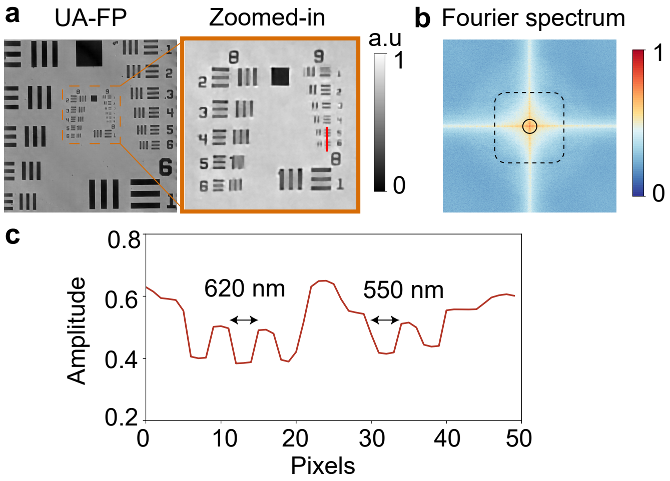

Figure S1. (a) Recovery amplitude-type resolution target result, (b) Fourier spectrum, and (c) profile map of the target line. The solid and dashed boxes represent objective lens NA and the maximum illumination NA.

The recovered pupil aberrations are depicted in Fig. S2a. The bar graph on the right side of Fig. S2a shows the coefficients of the first 15 order Zernike polynomials obtained using UA-FP . This analysis reveals that the system's aberrations are predominantly characterized by 5th-order $45°$ astigmatism and 11th-order spherical aberration. Fig. S2b illustrates the correction of the misaligned LED array, with the evolution of the $\boldsymbol{\theta}_{\mathrm{illum}}$ parameters during optimization shown in Fig. S2c. The convergence values from this experiment indicate that translation errors remain within $0.3\,\mathrm{mm}$ and rotation errors within $0.3°$, resulting in practically unnoticeable misalignment.

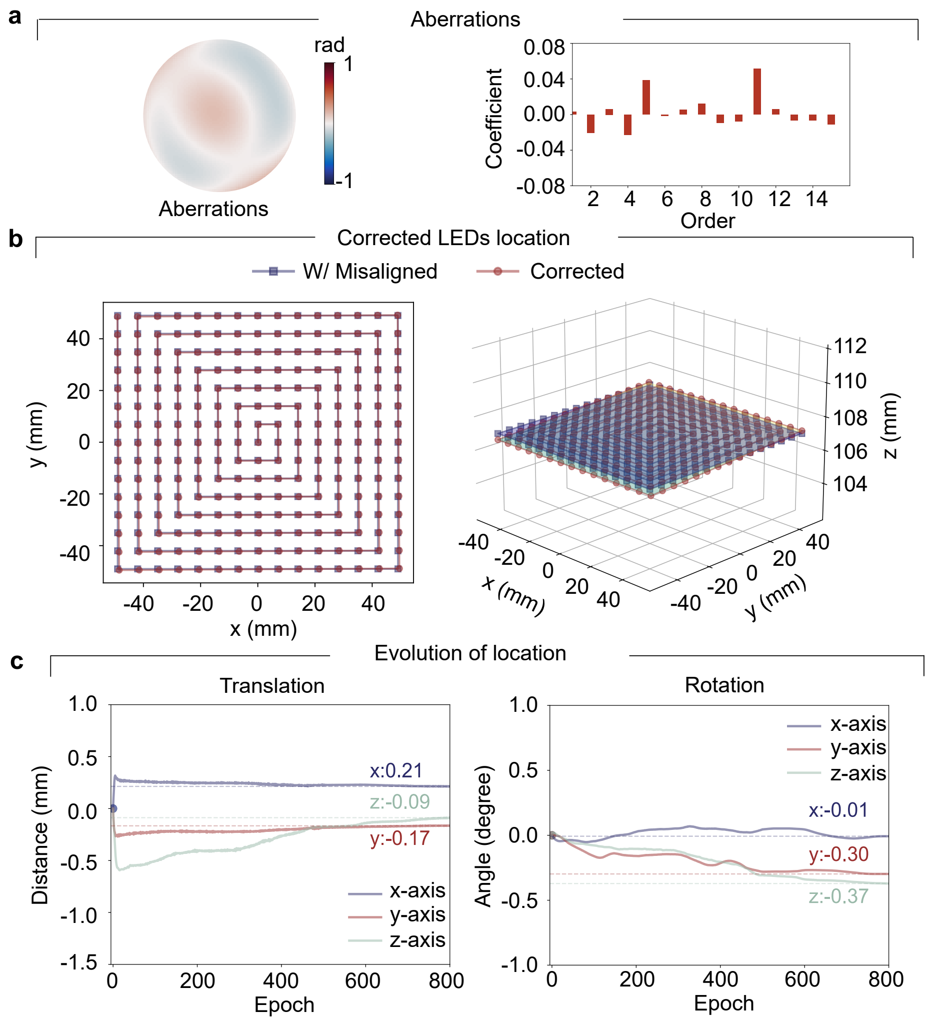

Figure S2. (a) Reconstruction of system parameters including aberrations and corresponding Zernike coefficients, and (b) 2D and 3D views of LED array location after correction. (c) Evolution of $\boldsymbol{\theta}_{\text{illum}}$ parameter during the optimization.

S2. Results with open-source datasets

We also evaluated the proposed UA-FP method using three open-source datasets. Dataset 1 is an amplitude-only USAF resolution chart[1], dataset 2 is a blood smear bio-sample[1], and dataset 3 is another amplitude-based USAF resolution chart[2]. The parameters of the imaging systems for each dataset are detailed in Table S1.

Table S1. Parameters of the open-source datasets.

	Objective lens	Sample-lens distance	Sensor pitch	Wave-length	Used LED number	LED gap
dataset 1[1]	2×(NA=0.1)	90.88 mm	3.69 µm	630 nm	15 × 15	4 mm
dataset 2[1]	2×(NA=0.1)	90.88 mm	3.69 µm	532 nm	15 × 15	4 mm
dataset 3[2]	4×(NA=0.1)	87.5 mm	6.5 µm	626 nm	21 × 21	2.5 mm

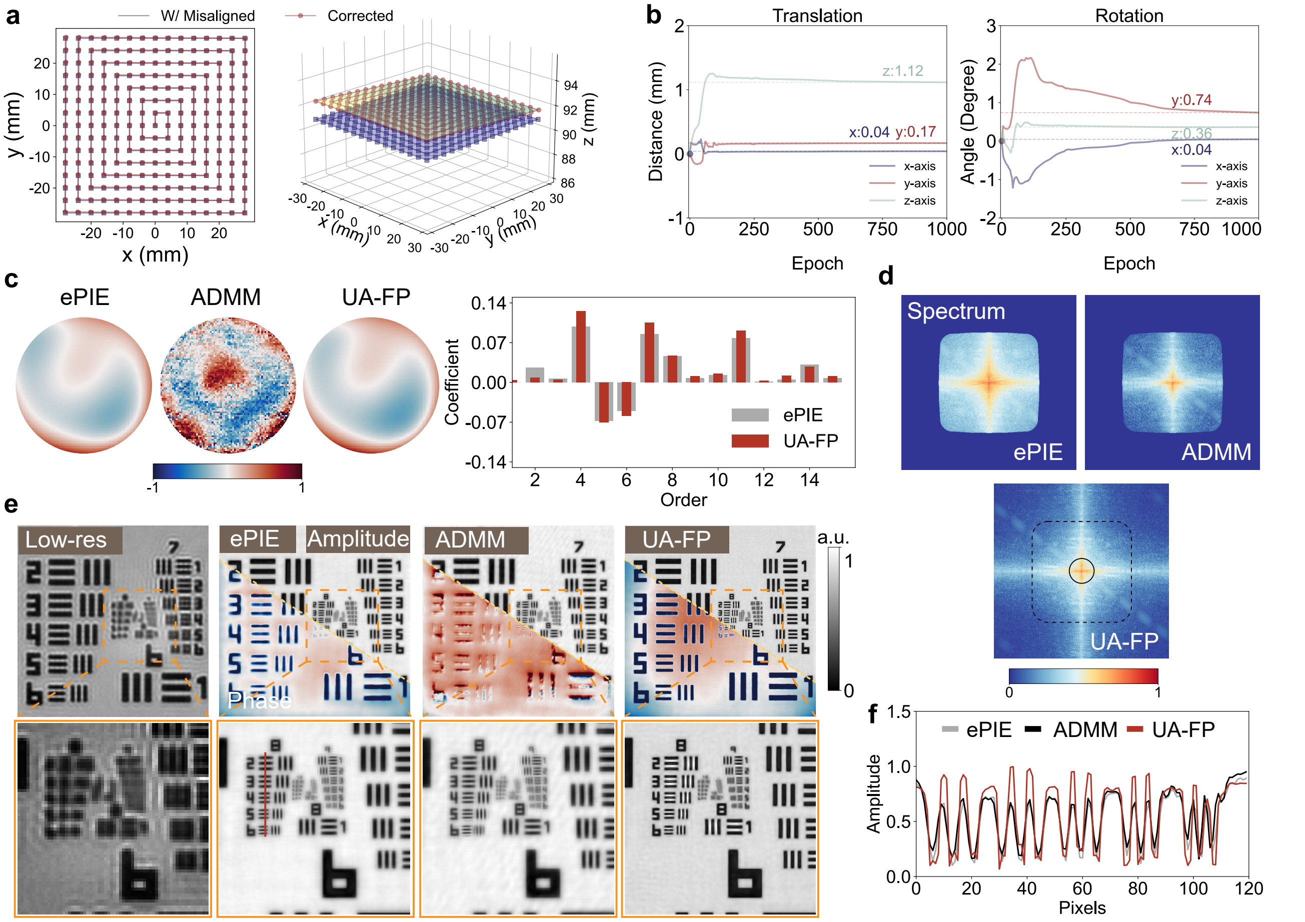

Figure S3. Recovery USAF result from open-source dataset 1[1]. (a) 2D and 3D views of the LED array after correcting misalignment. (b) Evolution of $\boldsymbol{\theta}_{\text{illum}}$ parameters during the optimization. (c) Reconstructed pupil function and (d) Fourier spectrum with different methods. (e) Reconstructed complex fields and (f) the line plot comparison.

Datasets 1 and 2 were obtained using the same well-aligned FP system with high-quality data[1], while Dataset 3 was captured using an even better-aligned FP system[2]. These datasets are employed to validate the performance of the UA-FP method across different imaging systems, each presenting unique uncertainties.

Fig. S3 presents the imaging results obtained from dataset 1[1]. Fig. S3a shows the LED array locations before and after correction, with the evolution of the $\boldsymbol{\theta}_{\text{illum}}$ parameters shown in Fig. S3b. The translation parameters for the x and y axes remain below $200\,\mu\mathrm{m}$, which is quite small compared to the LED pitch of $4\,\mathrm{mm}$. However, a significant deviation is observed along the z axis, with the distance corrected from the premeasured $90.88\,\mathrm{mm}$ to $92\,\mathrm{mm}$. Achieving this sub-millimeter precision requires advanced mechanical equipment and careful fine-tuning. The pupil function and Fourier spectrum are shown in Fig. S3c and d. For the reconstructed pupil, the ADMM[3] method reveals speckle-like points, as it models the pupil as a matrix rather than using Zernike coefficients. In contrast, ePIE[4] and UA-FP exhibit comparable aberration profiles, as demonstrated by the first 15 orders of Zernike coefficients in the bar graph on the right side of Fig. S3c. This consistency confirms the accuracy of the pupil function recovered by the proposed UA-FP. The Fourier spectrum in Fig. S3d is restricted by the synthesized aperture in conventional ePIE and ADMM approaches. In contrast, UA-FP achieves a computational resolution limit that extends the spectrum beyond these constraints. Fig. S3e compares the image reconstructions. While the zoomed-in images indicate similar resolution across all methods, as dictated by the maximum NA of illumination, both ePIE and ADMM reconstructions exhibit a blur effect. In contrast, the UA-FP reconstruction demonstrates higher contrast, particularly at the target boundaries. For better comparison, the profile of the group 8 lines is shown in Fig. S3f, where the UA-FP method clearly achieves superior contrast compared to the other methods.

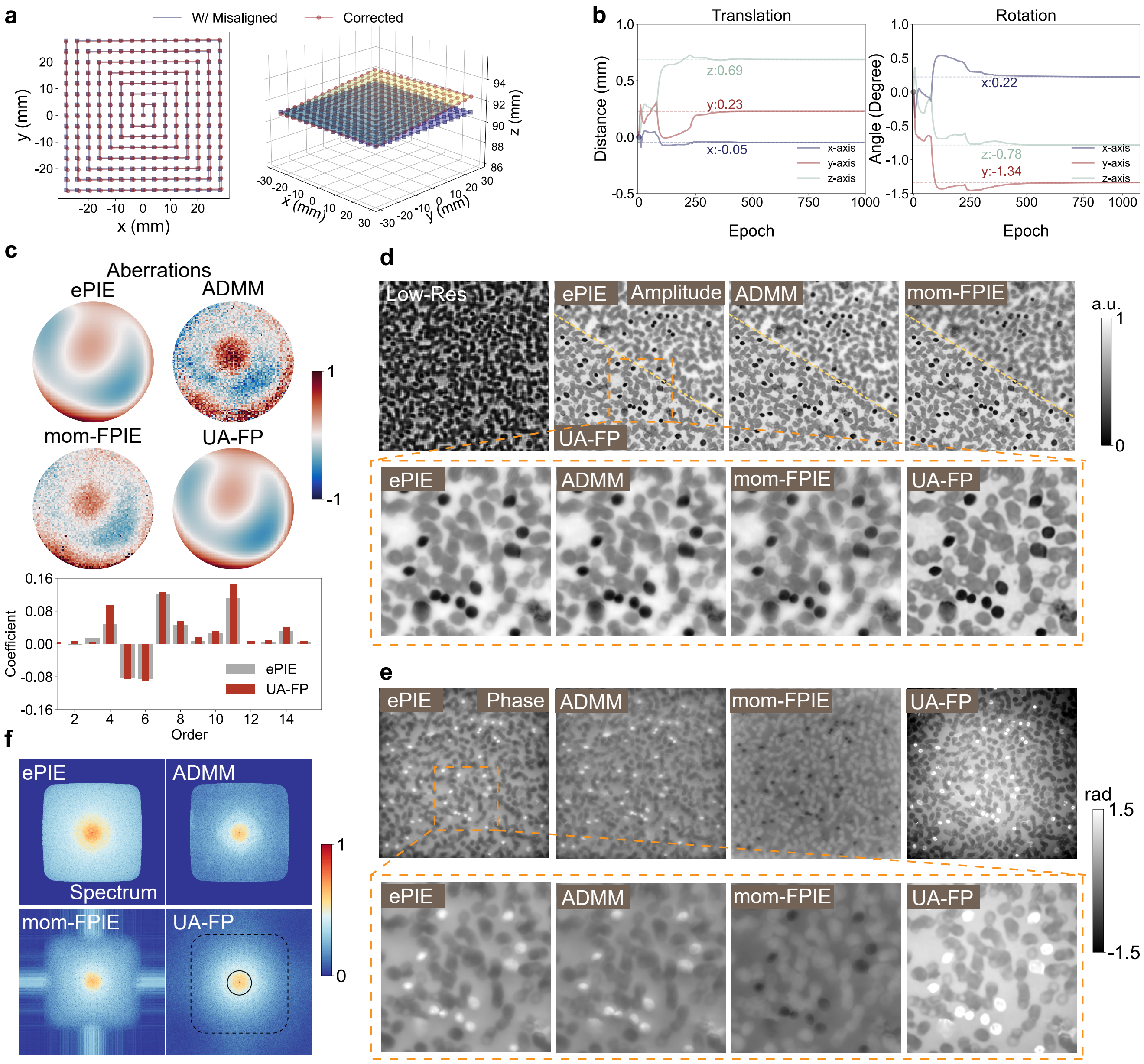

Figure S4. Recovery blood smear result from open-source dataset 2[1]. (a) 2D and 3D views of the LED array after correcting misalignment. (b) Evolution of θ_{illum} parameters during the optimization. (c) Reconstruction aberrations and Zernike coefficients of modes, (d) raw low resolution image and reconstruction amplitude, (e) phase, and (f) Fourier spectrum by different methods.

We also validate the imaging performance of UA-FP on a biological sample using dataset 2[1], as shown in Fig. S4. Fig. S4a demonstrates the misalignment correction, which differs slightly from that in Fig. S3a. The reconstructed aberrations in Fig. S4c are similar to those in Fig. S3d as both datasets were captured using the same imaging system. The slight differences in the estimated misalignment and aberrations represented by Zernike coefficients may be influenced by the sample's thickness. With uncertainty estimation, the reconstructed target is presented in Fig. S4d and e. Clearly, compared to ePIE[4], ADMM[3], and mom-FPIE[5], the reconstructions from UA-FP display finer details and higher contrast. Additionally, for the reconstructed phase, illustrated in Fig. S4e, ePIE, ADMM, and mom-FPIE show blurry results, with blood cells barely distinguishable from the background. In contrast, UA-FP presents clear cells that are easily distinguishable. This superior performance stems from UA-FP 's ability to distinguish the sources of aberrations by effectively aligning the LED array and characterizing

optical elements. The recovered aberrations are guaranteed to originate from the optical system rather than being influenced by LED misalignment, resulting in enhanced image quality. Notably, the hollow cell shape is observed in both the amplitude and phase reconstructions of UA-FP , reflecting the higher resolution achieved by UA-FP . This is attributed to UA-FP 's "computational resolution limit," which extends the Fourier spectrum beyond the physical diffraction limit of the optical system, as shown in Fig. S4f.

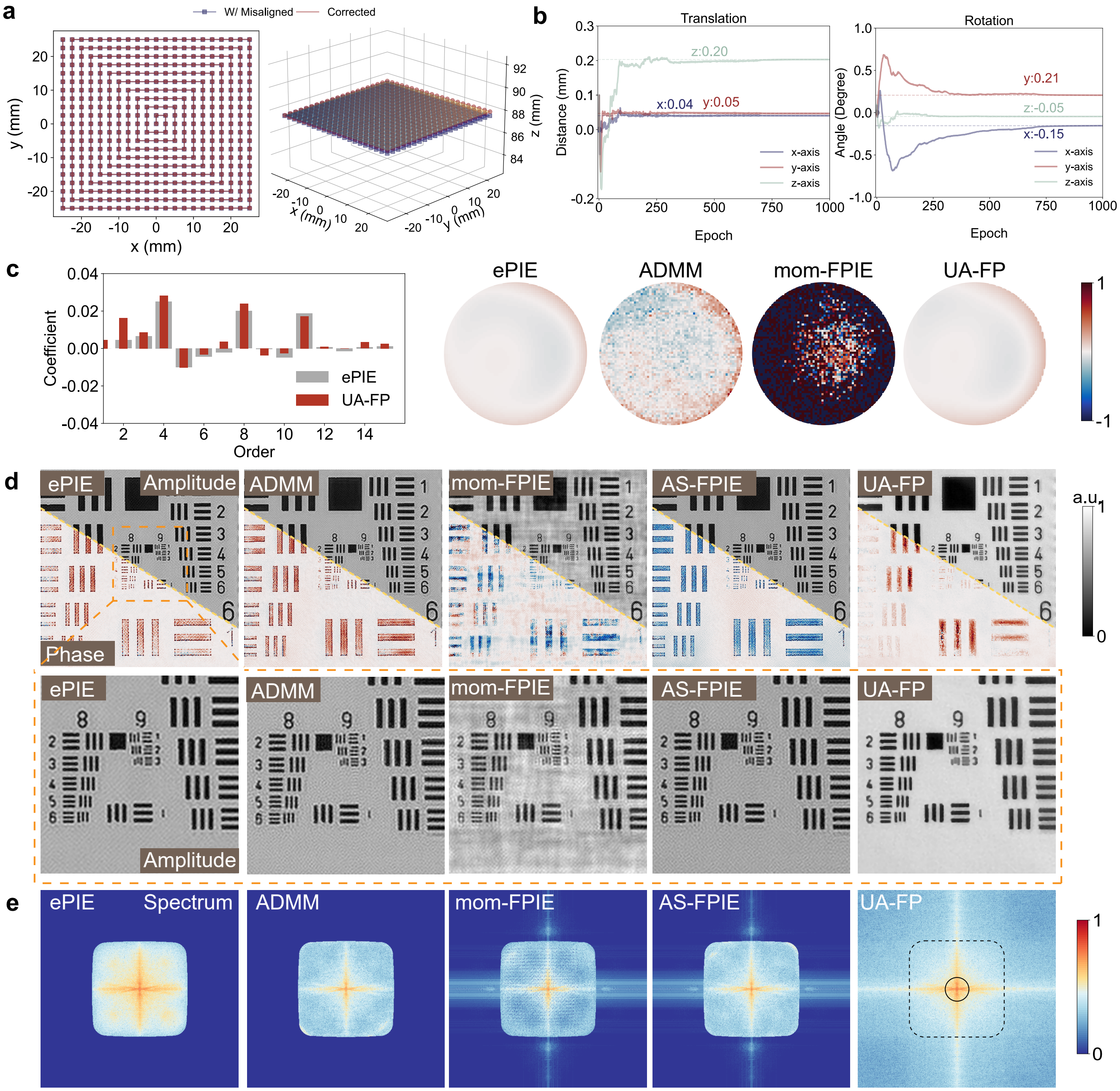

Figure S5. Recovery USAF result from open-source dataset 3[2]. (a) 2D and 3D views of the LED array after correcting misalignment. (b) Evolution of θ_{illum} parameters during the optimization. (c) Reconstruction aberrations, (d) USAF target, and (e) Fourier spectrum by different methods. Adaptive-Step FPIE (AS-FPIE)[6].

We further validate UA-FP using dataset 3, another open-source dataset obtained from a well-aligned system[2]. The estimated misalignment is displayed in Fig. S5a and the evolution of the θ_{illum} parameters in Fig. S5b shows that the translation and rotation parameters are subtle, confirming that the system is well aligned. When the

misalignment of the LED array is minimal, the aberration recovered by ePIE is quite accurate and can serve as a reference. As expected, the aberration reconstructed by UA-FP closely matches the ePIE reconstruction, as shown in Fig. S5c. Consequently, the reconstructions from all methods exhibit similar image quality and resolution, as demonstrated in Fig. S5d.

S3. Feature regularization performance

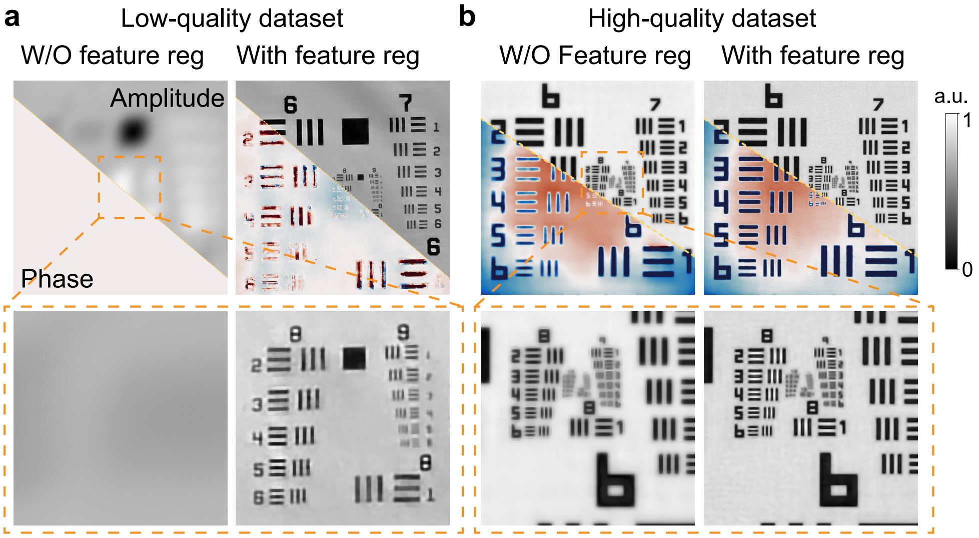

Figure S6. Reconstruction results without (W/O) and with the feature-extraction regularization using UA-FP . Reconstruction is performed on (a) low quality dataset and (b) open-source dataset 1 with relatively high quality measurements[1].

$$\underset{\boldsymbol{u}_{\text{obj}},\, \boldsymbol{\theta}_{\text{sys}},\, \boldsymbol{\theta}_{\text{illum}}}{\text{argmin}} \quad \sum_{n=1}^{N} \left\| f(\boldsymbol{u}_{\text{obj}},\, \boldsymbol{\theta}_{\text{sys}},\, \boldsymbol{\theta}_{\text{illum}}) - \boldsymbol{y}_n \right\|^2 + \beta_1 \mathcal{R}_{TV}(\boldsymbol{u}_{\text{obj}}) + \beta_2 \sum_{n=1}^{N} \left| \Phi[f(\boldsymbol{u}_{\text{obj}},\, \boldsymbol{\theta}_{\text{sys}},\, \boldsymbol{\theta}_{\text{illum}})] - \Phi[\boldsymbol{y}_n] \right| \tag{S1}$$

Figure S6 illustrates the benefits of the feature-extraction regularization $\Phi(\cdot)$ in the design of the loss function of Eq. S1. The specific implementation of $\Phi(\cdot)$ involves convolving images with the kernels $[0, -1, 1]$ and $[0, -1, 1]^T$ along the x and y axes, respectively[7]. Without $\Phi(\cdot)$, the method fails to recover the target accurately, producing artifacts due to poor data quality, as shown in Fig. S6a. However, with feature extraction, the target can be reconstructed from implicit data features, resulting in successful recovery even with low quality data. Using high-quality open-source dataset[1], $\Phi(\cdot)$ further improves imaging quality, as demonstrated in Fig. S6b. Without $\Phi(\cdot)$, the recovered USAF target appears blurry, whereas UA-FP , with the inclusion of $\Phi(\cdot)$, yields images with higher contrast and sharper focus.

S4. Anti-noise performance

This numerical analysis illustrates the anti-noise performance of the proposed UA-FP method. In this simulation, Gaussian noise $\varepsilon_{\text{bright}}$ is applied to the bright-field measurements, while Poisson noise $\varepsilon_{\text{dark}}$ is introduced to the dark-field measurements. The Gaussian noise $\varepsilon_{\text{bright}}$ is written in Eq. S2.

$$\varepsilon_{\text{bright}} = \mathcal{N}_{\mathcal{G}}(\mu, \sigma^2) \tag{S2}$$

Here, $\mathcal{N}_{\mathcal{G}}$ represents the Gaussian distribution, and μ and σ denote the mean value and standard deviation. The final bright-field noisy images $\boldsymbol{y}_n^{\text{bright}}$ are expressed as:

$$\begin{aligned} \boldsymbol{y}_n^{\text{bright}} &= f_N \circ f\left(\boldsymbol{u}_{\text{obj}}, \boldsymbol{\theta}\right) \\ &= f\left(\boldsymbol{u}_{\text{obj}}, \boldsymbol{\theta}\right) + \varepsilon_{\text{bright}} \end{aligned} \tag{S3}$$

For different Gaussian noise levels, the mean value μ is set to 0, and σ is obtained by multiplying a ratio with the maximum value in the low-resolution images $\boldsymbol{y}_n$. In this anti-noise simulation, three Gaussian noise ratios are selected: 0.05, 0.1, and 0.2.

For Poisson noise, levels 1 through 4 correspond to the number of photons detected per pixel: 80, 40, 20, and 1 photon, respectively. The detailed calculation for dark-field noisy images $\boldsymbol{y}_n^{\text{dark}}$ is as follows:

$$\gamma = \frac{p_n \times h \times w}{\sum f\left(\boldsymbol{u}_{\text{obj}}, \boldsymbol{\theta}\right)}, \quad \boldsymbol{y}_n^{\text{dark}} = f_N \circ f\left(\boldsymbol{u}_{\text{obj}}, \boldsymbol{\theta}\right) = \frac{\mathcal{P}(f\left(\boldsymbol{u}_{\text{obj}}, \boldsymbol{\theta}\right) \times \gamma)}{\gamma} \tag{S4}$$

Here, p_n represents the number of photons detected per pixel, while h and w denote the height and width of the low-resolution image. $\sum \boldsymbol{y}_n$ refers to the intensity sum of a low-resolution image. $\mathcal{P}(\cdot)$ is the Poisson distribution. It is important to note that in our simulation, any negative values in the noise-added measurements are set to 0, as negative values do not occur in real optical experiments. Table S2 provides the detailed metric values for the anti-noise performance of various methods. As seen from the results, for all methods, the PSNR and SSIM of the reconstructed images decrease as the noise level increases. This decline is particularly pronounced for traditional methods like ePIE, mom-FPIE, and mc-FPIE. In contrast, the UA-FP method demonstrates superior anti-noise performance, with minimal reduction in reconstruction quality. This advantage is attributed to our differentiable FP technology, which leverages modern numerical optimization and an accurate forward imaging model, surpassing traditional recovery methods and offering enhanced resilience to noise.

Table S2. Anti-noise performance of various methods.

Gaussian	Poisson	Average PSNR (dB) and SSIM				
		ePIE[4]	ADMM[3]	mom-FPIE[5]	mc-FPIE[8]	UA-FP
0.05	Level 4	15.81/0.7895	17.54/0.8016	16.24/0.7605	5.46/0.0911	18.93↑/0.8407↑
	Level 3	18.31/0.8558	21.45↑/0.8833	19.27/0.8824	9.36/0.2921	19.87/0.8976↑
	Level 2	18.44/0.8600	21.75↑/0.8854	19.88/0.8933	9.36/0.3295	19.45/0.8981↑
	Level 1	18.32/0.8610	21.68↑/0.8871	19.94/0.8932	10.24/0.3594	19.01/0.8928↑
0.1	Level 4	15.70/0.7694	16.41/0.7756	14.19/0.7395	5.45/0.0930	18.23↑/0.8293↑
	Level 3	16.80/0.8085	18.15/0.8339	16.22/0.8388	8.82/0.2751	19.90↑/0.8929↑
	Level 2	16.56/0.8070	18.28/0.8347	15.25/0.8394	9.55/0.2982	19.21↑/0.8917↑
	Level 1	16.76/0.8054	18.17/0.8337	15.94/0.8428	9.91/0.3198	18.62↑/0.8870 ↑
0.2	Level 4	13.88/0.6964	15.28/0.7230	11.31/0.6744	5.41/0.0874	17.33↑/0.8124↑
	Level 3	13.47/0.6902	16.09/0.7526	11.95/0.7321	8.61/0.2394	19.20↑/0.8797↑
	Level 2	13.23/0.6807	16.15/0.7468	12.25/0.7369	9.23/0.2630	18.58↑/0.8795↑
	Level 1	13.42/0.6747	15.91/0.7455	12.25/0.7357	9.75/0.2809	18.43↑/0.8768 ↑

S5. Verification of the interplay between the pupil function and LED array location

In the UA-FP method, we adopt a shift-invariant imaging model to build the forward model. However, in this shift-invariant imaging model, the misalignment of the LED array can also lead to an inaccurate pupil reconstruction since it results from the combined effect of the LED array and the optical system. Based on Equations S5 and S6, the LED location affects the shifting of the coherent transfer function (CTF) in the frequency domain, which is closely related to the aberration function.

$$\text{CTF}_{\nabla} = \frac{1}{1 + \exp\left\{\left[(k_x - k_x(\boldsymbol{\theta}_{\text{illum}}))^2 + (k_y - k_y(\boldsymbol{\theta}_{\text{illum}}))^2\right] - (\text{NA} \times k_0)^2\right\}} \tag{S5}$$

$$p(\boldsymbol{\theta}_{\text{sys}}) = \text{CTF}_{\nabla} \times \prod_{n,m=0}^{n,m} \phi(\boldsymbol{\theta}_n^m) \tag{S6}$$

$\phi(\boldsymbol{\theta}_n^m)$ represents the aberration function composed of Zernike Polynomials. Since $\boldsymbol{\theta}_{\text{illum}}$ and $\boldsymbol{\theta}_{\text{sys}}$ are connected through a multiplication operation, they do not function as independent variables. It is evident that misaligned LED

locations would lead to errors in CTF shifting, which consequently results in the recovered aberration becoming a combination of LED location errors and true system aberrations.

To illustrate this interaction, we compare the reconstructed aberration of the experimental data with and without LED alignment, as shown in Figure S7. The decision to align or not align the LED arrays significantly impacts the final recovered Zernike coefficients, confirming that LED location affects the pupil function characterization.

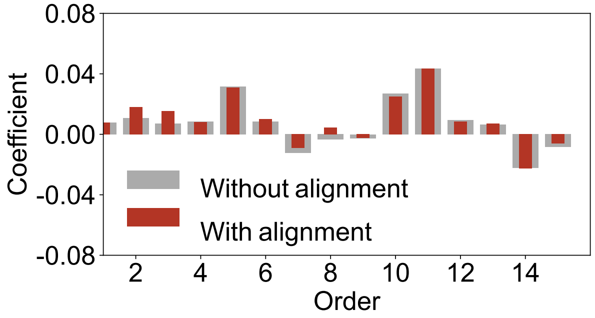

Figure S7. Reconstructed Zernike coefficients with and without LED array alignment.

References

1. Song, P. & Zheng, G. Fourier-ptychography. https://github.com/SmartImagingLabUConn/{Fourier}-Ptychography (2020).
2. Zuo, C. Smart computational imaging (sci) laboratory. https://www.scilaboratory.com (2016).
3. Wang, A. Y. et al. Fourier ptychographic microscopy via alternating direction method of multipliers. *Cells* **11**, 1512, doi: 10.3390/cells11091512 (2022).
4. Ou, X. Z., Zheng, G. A. & Yang, C. Embedded pupil function recovery for Fourier ptychographic microscopy. *Optics Express* **22**, 4960–4792, doi: 10.1364/oe.22.004960 (2014).
5. Maiden, A., Johnson, D. & Li, P. Further improvements to the ptychographical iterative engine. *Optica* **4**, 736–745, doi: 10.1364/OPTICA.4.000736 (2017).
6. Zuo, C., Sun, J. & Chen, Q. Adaptive step-size strategy for noise-robust Fourier ptychographic microscopy. *Optics Express* **24**, 20724, doi: 10.1364/oe.24.020724 (2016).
7. Zhang, S. H., Berendschot, T. T. J. M. & Zhou, J. H. Elfpie: an error-laxity Fourier ptychographic iterative engine. *Signal Processing* **210**, 109088, doi: https://doi.org/10.1016/j.sigpro.2023.109088 (2023).
8. Zhou, A. et al. Fast and robust misalignment correction of Fourier ptychographic microscopy for full field of view reconstruction. *Optics Express* **26**, 23661–23674, doi: 10.1364/oe.26.023661 (2018).
